# Effectiveness of Decompressive Hemicraniectomy to Treat a Life-Threatening Cerebral Fat Embolism

**DOI:** 10.1155/2019/2708734

**Published:** 2019-02-28

**Authors:** Charlène Couturier, Guillaume Dupont, François Vassal, Claire Boutet, Jérôme Morel

**Affiliations:** ^1^Anesthesia and Intensive Care Medicine Department (MD), Saint Etienne University Hospital Center, Avenue Albert Raimond, 42270 Saint-Priest-En-Jarez, France; ^2^Neurosurgery Department, Saint Etienne University Hospital Center, Avenue Albert Raimond, 42270 Saint-Priest-En-Jarez, France; ^3^Radiology Department, Saint Etienne University Hospital Center, Avenue Albert Raimond, 42270 Saint-Priest-En-Jarez, France; ^4^Anesthesia and Intensive Care Medicine Department, Saint Etienne University Hospital Center, Avenue Albert Raimond, 42270 Saint-Priest-En-Jarez, France

## Abstract

**Background and Importance:**

Cerebral fat embolism (CFE) occurs mainly after long-bone fractures. Often reducing to minor neurological disorders as confusion, it can sometimes cause more severe consequences such as coma or even death. While CFE has been described for several years, there is no consensual treatment.

**Clinical Presentation:**

We report the case of a 15-year-old girl with a severe cerebral fat embolism secondary to a longboard fall with a femur fracture. She developed in less than 4 hours a coma. On day 4, she lost her brainstem reflexes with a clinical condition close to brain death, with a very high intracranial pressure (ICP) value above 75 mmgH at worst. She was treated as having a trauma brain injury based on ICP control with a decompressive hemicraniectomy. She recovered in some weeks, allowing discharge to a post ICU rehabilitation center, one month after admission.

**Conclusion:**

We report a severe case of cerebral fat embolism with good outcome. It was managed as a trauma brain injury. We emphasize the neurological management based on ICP and discuss the position of hemicraniectomy.

## 1. Introduction

Cerebral fat embolism (CFE) is a rare complication of long bone fracture with a mortality rate between 7.4% and 36 % [1, 2]. CFE is difficult to diagnose and the physiopathology is not well understood. Symptoms are delayed from the initial insult; 24 from 72 hours is classically described [3]. Shortest delay is recognized as a pejorative prognostic factor [4].

There is no consensus concerning the treatment. In some serious cases, management of intracranial pressure has been proposed with mixed results [1, 5, 6].

We report the case of a 15-year-old woman with an unusual presentation of CFE. The symptoms appeared a few hours after the initial trauma and worsened into a life-threatening intracranial hypertension (ICH). Despite the initial severity, the patient recovered almost completely after some weeks. We discuss the management of ICH and the position of hemicraniectomy in this situation.

## 2. Case Presentation

A 15-year-old woman with no medical history was admitted to our intensive care unit (ICU) a few hours after a longboard fall without initial loss of consciousness or head trauma. The patient was not able to walk and she had to be transported to find help. When the medical team arrived, she was conscious, Glasgow coma scale (GCS) of 15, without hemodynamic or respiratory instability and with a left femur fracture. During the medical transport, she received analgesics medications and immobilization after the reduction of the fracture. The initial body CT scanner, performed 3 hours after the trauma, found a left femur fracture and an anterior left pneumothorax, without cerebral lesions.

**Figure 1:**
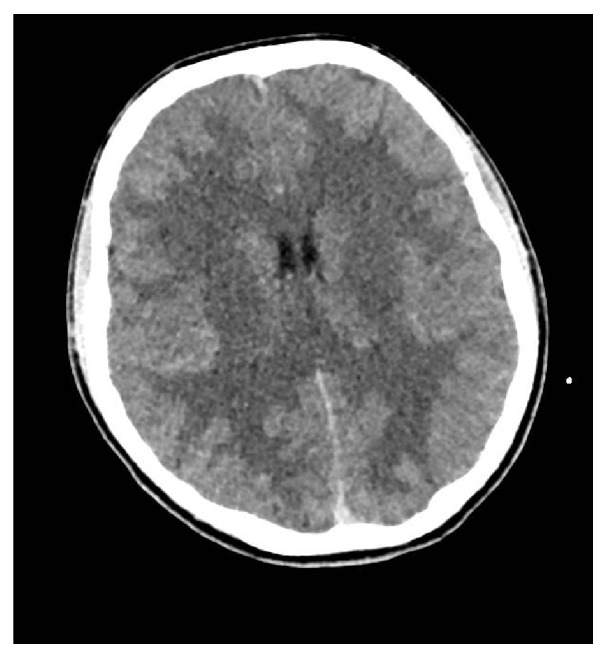
Cranial computed tomography (CT) scan performed at the end of the surgery: brain swelling.

She presented secondarily a neurologic status impairment with a GCS of 11, initially attributed to an excess of analgesic therapy. Anyway, she was operated with a left femoral nailing during which a prolongated hypotension without hypovolemia or other obvious causes occurred. At the end of the surgery, 7 hours after the initial injury a new brain scan was performed. It showed the appearance of a cerebral swelling (Figure 1).

Postoperatively, she was admitted to the ICU because of consciousness disorders requiring a drug induced coma to permit a mechanical ventilation. A cerebral fat embolism was rapidly suspected. Despite a hemodynamic stability and a normality of the PaCO_2_, the transcranial Doppler ultrasound found a bilateral high pulsatility index at 2.2 and low end-diastolic flow velocity below 20 cm/s. These Doppler profiles led us to suspect an intracranial hypertension. A new brain CT scan, performed 16 hours after the trauma, confirmed a diffuse major cerebral edema. No other organ dysfunctions, rash, or petechiae were noticed.

**Figure 2:**
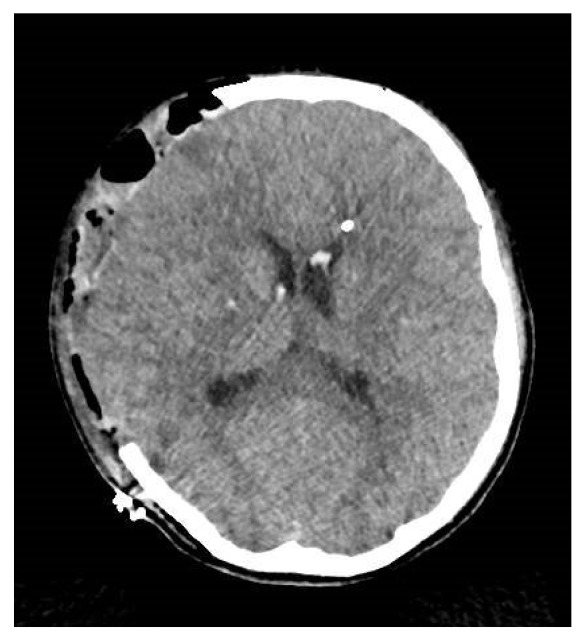
Cranial CT scan after decompressive hemicraniectomy, insertion of external ventricular derivation, and intracranial pressure catheter: cerebral swelling; hematoma on the right frontotemporal-parietal craniectomy.

The patient was managed as a severe brain injury. An intracranial pressure catheter was inserted and found a very high intracranial pressure (ICP) of 75 mmHg. Despite a maximal medical treatment including osmotherapy, hypothermia, barbiturate sedation, and use of neuromuscular-blocking drugs, the ICP remained above 35 mmHg. Twenty-two hours after admission, the patient presented a bilateral reactive pupillary enlargement. The neurosurgeon immediately performed a decompressive right fronto-temporo-parietal hemicraniectomy. Afterwards, the intracranial pressure remained between 20 and 25mmHg and an external ventricular derivation was inserted. A control brain CT scan was performed (Figure 2).

**Figure 3:**
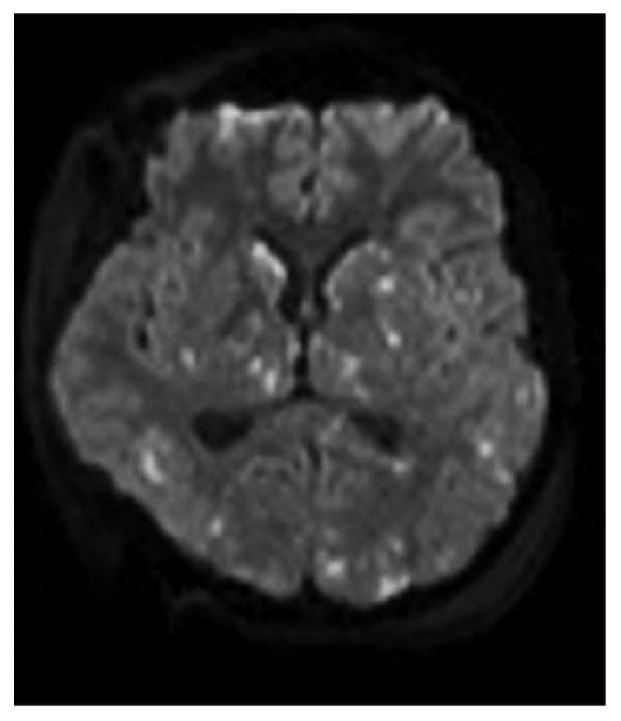
Magnetic resonance imaging, on diffusion-weighted imaging (DWI) sequences, performed 4 days after the trauma showing punctuate hyperintense foci: Starfield pattern.

On the fourth day, the patient presented signs of brainstem injury with a bilateral unreactive mydriasis and loss of oculocardiac reflex despite the normalization of the ICP under 20 mmHg. The patient was still under sedative drugs. A cerebral magnetic resonance imaging (MRI) was carried out. T2-weighted, fluid-attenuated inversion recovery, and diffusion-weighted magnetic resonance imaging showed diffuse punctate hyperintense foci of restricted diffusion in both cerebral and cerebellar hemispheres, with susceptibility artifacts on susceptibility weighted MRI sequences in keeping with petechial hemorrhagic foci, in a starfield pattern (Figure 3).

**Figure 4:**
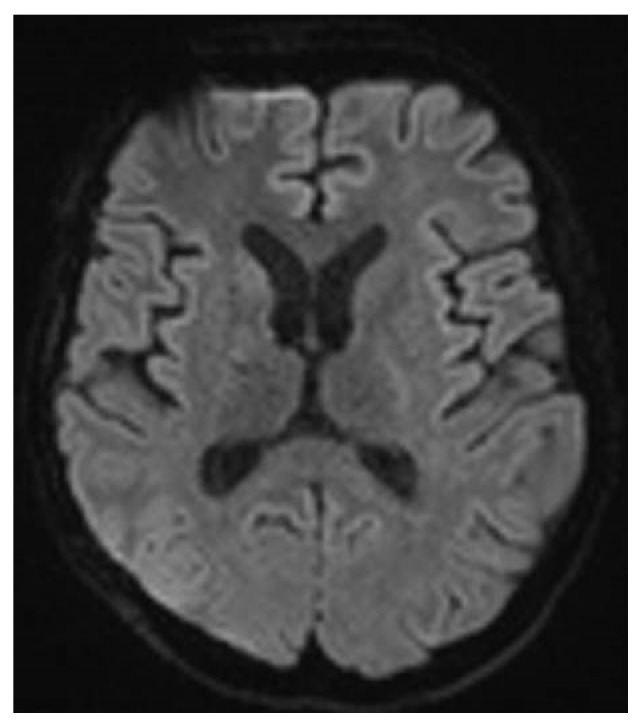
Magnetic resonance imaging at 3 months on DWI sequences showing a regression of punctate hypersignal lesions.

Progressively, her consciousness improved with a GCS 9 (M4, V1, E4) ten days after the trauma. One month after her admission she was discharged to a rehabilitation center with a GCS 11 (M6 V1 E4). At two months, she was still improving with a GCS 15, but with persistence of few cognitive disorders evaluated by brain trauma scales: Montreal Cognitive Assessment scale at 26/30; Ranchos scale at VII. The cerebral MRI at three months showed a regression of the multiple punctate hypersignal lesions on diffusion sequences and a disappearance of the hypersignals FLAIR and diffusion of the striatum (Figure 4). At 6 months after the trauma, she could reintegrate her school. She kept only some headaches and an asthenia one year later.

We have the patient's consent for publishing this case report. The ethic committee approval was not required according to French legislation.

## 3. Discussion

Cerebral fat embolism is a rare pathology, described in 10% of fat embolism syndrome [7]. CFE is difficult to diagnose and with no specific treatment. The physiopathology is still unclear. When there is not a patent foramen ovale, the hypothesis of a pulmonary arterioveinous communication, pulmonary lymphatic resorption, or even a toxicity by fat embolism metabolites [4, 8] is proposed.

Cases reported in the literature described various neurological symptomatologies ranging from confusion to severe encephalopathy [1]. Overall mortality is 7.4 % [1]. Good outcomes (intact or mild disability) are reported in most of survivors (72.2 %) [1]. Coma at the presentation worsens the prognosis with a good outcome in 57.6 % [1]. The free interval between the trauma and the first neurologic signs is usually between 24 and 72 hours [3]. In most of the cases, patients were treated with a supportive treatment.

Our patient developed in only a few hours a severe cerebral injury due to a CFE evolving toward brainstem lesions. We chose to combine medical treatment and decompressive hemicraniectomy as recommended in some studies about brain injury management [9–11]. Finally, despite the severity of cerebral clinical state, she recovered fully in few months without irreversible brain damages and or physical handicap.

Decompressive craniectomy is recommended for patients with malignant edema in acute ischemic stroke and has been shown to reduce mortality and improve neurologic outcomes [9, 10]. For the patients with severe traumatic brain injury the efficacy of the craniectomy to control the ICP is clearly demonstrated but the link with the neurologic outcome is less evident [12]. Overall, this technique is currently recommended in the latest traumatic brain injury guidelines [10]. The effectiveness of craniectomy to manage intracranial hypertension in the other situations such as subarachnoid hemorrhage is still debated [13]. In life-threatening cerebral fat embolism, decompressive craniectomy was documented in one case with poor outcomes [1] and the measurement of intracranial pressure in only three cases [5, 6, 14]. In two of these cases, there is no description of the severity and the management of the intracranial hypertension [5, 6]. The third had poor outcome with vegetative coma after only a medical neurologic treatment [14]. To our knowledge, this is the first patient with intracranial hypertension caused by cerebral fat embolism successfully treated using a medical and surgical treatment.

We propose that the neurological management of these patients could be based on trauma brain injury guidelines including ICP monitoring and surgical control of refractory intracranial hypertension.

For conclusion, in case of severe cerebral edema, a strategy based on cerebral perfusion preservation can be discussed. Our neurologic management was the unique point that differed from the other cases with the same severity and clinical presentation. This case underlines the interest of an aggressive neurologic treatment based on ICP monitoring and a full commitment in view of these reversible injuries. A large cohort is warranted to validate this strategy.
